# The Impact of Receiving Pretravel Health Advice on the Prevention of Hajj-Related Illnesses Among Australian Pilgrims: Cohort Study

**DOI:** 10.2196/10959

**Published:** 2020-07-14

**Authors:** Amani S Alqahtani, Saeed A Alsharif, Mohammad A Garnan, Mohamed Tashani, Nasser F BinDhim, Anita E Heywood, Robert Booy, Kerrie E Wiley, Harunor Rashid, Hajj Research Group

**Affiliations:** 1 Saudi Food and Drug Authority Riyadh Saudi Arabia; 2 School of Public Health The University of Sydney Sydney Australia; 3 National Centre for Immunisation Research and Surveillance of Vaccine Preventable Diseases The Children’s Hospital at Westmead Sydney Australia; 4 Ministry of Health Taif Saudi Arabia; 5 Ministry of Health Abha Saudi Arabia; 6 The Discipline of Child and Adolescent Health Sydney Medical School University of Sydney Sydney Australia; 7 School of Public Health and Community Medicine The University of New South Wales Sydney Australia; 8 Marie Bashir Institute for Infectious Diseases and Biosecurity Sydney Australia; 9 WHO Collaborating Centre for Mass Gatherings and High Consequence/High Visibility Events Flinders University Adelaide Australia; 10 See Acknowledgments

**Keywords:** Hajj, health behavior, mass gathering, pretravel health advice, travelers

## Abstract

**Background:**

Pretravel health advice can play a crucial role in improving both travelers’ awareness about disease risk and compliance with preventive measures. General practitioners (GPs) and the internet have been reported internationally to be the main sources of health advice for travelers to non–mass gathering (MG) destinations. However, few studies have attempted to investigate the sources of health advice among travelers to MGs including the Hajj pilgrimage, and none of these studies further investigated the impact of pretravel advice on pilgrims’ health behaviors.

**Objective:**

The objective of this study was to investigate the impact of the source of pretravel health advice (from GPs and specialized Hajj travel agents) on Hajj pilgrims’ awareness of and compliance with health recommendations, and the incidence of Hajj-associated illnesses.

**Methods:**

A prospective cohort study (before and during Hajj) was conducted among Australian pilgrims aged ≥18 years in 2015.

**Results:**

A total of 421 pilgrims participated prior to Hajj, and 391 (93%) provided follow-up data during Hajj. All participants obtained pretravel health advice from one or more sources, with Hajj travel agents (46%) and general practitioners (GPs; 40%) the most commonly reported sources. In total, 288 (74%) participants reported Hajj-related symptoms, of which 86% (248/288) were respiratory symptoms. Participants who obtained pretravel health advice from travel agents were more likely to be aware of the official Saudi recommendations (adjusted odds ratio [aOR] 2.1, 95% CI 1.2-3.8; *P*=.01), receive recommended vaccines before travel (aOR 2.4, 95% CI 1.4-3.9; *P*=.01), use hand sanitizers including soap (aOR 2.5, 95% CI 1.1-6.1; *P*=.03), and wash their hands after touching an ill person during Hajj (aOR 2.9, 95% CI 1.1-7.1; *P*=.01), compared to those who sought advice from GPs. However, neither advice from travel agents nor GPs was associated with a lower incidence of Hajj-related illnesses.

**Conclusions:**

Advice from travel agents appeared to be accessed by more travelers than that from GPs, and was associated with an increased likelihood of positive travel health behaviors.

## Introduction

As more and more people travel each year, the spread of infectious diseases via international travel presents an increasing challenge to disease control globally [1]. Travelers to mass gatherings (MGs) play a significant role in the spread of infectious diseases across international borders due to their unique travel patterns and behaviors [2,3]. An MG has been defined as an event involving large number of participants (≥1000 attendees) at a specific location for a specific purpose for a defined period of time [4]. Hajj pilgrimage to Makkah, Saudi Arabia, is a noteworthy example of an MG. With 2 to 3 million attendees from about 185 countries attending annually, it is considered to be one of the largest annual MGs in the world. The Ministry of Health (MoH) in Saudi Arabia requires that all pilgrims are vaccinated against meningococcal disease, and that pilgrims from endemic countries are vaccinated against polio and yellow fever (Textbox 1) [5,6]. Moreover, vaccines against influenza, pertussis, mumps, and measles are also recommended, as well as other infection control measures, including the use of face masks and hand hygiene. Health authorities in travelers’ countries of origin are also encouraged to provide health education to the pilgrims [5,6].

**Health recommendations and preventive measures for travelers to Saudi Arabia for Hajj 2016.**

**Compulsory vaccines**

- Quadrivalent meningococcal vaccine (ACYW135): Compulsory for all pilgrims. Administered not less than 10 days before arrival.
- Oral polio vaccine (OPV) or inactivated polio vaccine (IPV): Compulsory for pilgrims from endemic countries. Administered at least 4 weeks before arrival. Other pilgrims should remain up-to-date.
- Yellow fever vaccine: Compulsory for pilgrims from endemic countries or those transiting through endemic countries. Administered at least 10 days before arrival.

**Recommended vaccines**

- Seasonal influenza vaccine: Recommended for all, in particular at-risk pilgrims.
- Diphtheria vaccine: Remaining up to date.
- Pertussis vaccine: Remaining up to date.
- Measles vaccine: Remaining up to date.
- Mumps vaccine: Remaining up to date.
- Tetanus vaccine: Remaining up to date.

**Nonpharmaceutical measures**

- Wash hands with soap and water or disinfectant, especially after coughing and sneezing, after using the toilet, before handling and consuming food, and after touching animals.
- Use disposable tissues when coughing or sneezing and dispose of them afterwards in waste baskets.
- Avoid hand contact with the eyes, nose, and mouth.
- Wear face masks, especially in crowded places.
- Avoid direct contact with persons who appear to be ill with coughing, sneezing, expectorating, vomiting, diarrhea, and do not share personal belongings.
- Maintain good personal hygiene.
- Avoid contact with sick animals.
- Avoid drinking raw camel milk or camel urine or eating meat that has not been properly cooked.
- Take insect bite avoidance measures during daytime and nighttime hours to reduce the risk of infection with dengue and other mosquito-borne diseases.

**Health education**

- Health authorities in countries of origin are required to provide health information to pilgrims on infectious disease symptoms, transmission mode, and preventive measures.

Pretravel health advice can play a crucial role in improving both travelers’ awareness about disease risk and compliance with preventive measures. General practitioners (GPs) and the internet have been reported internationally as the main sources of health advice for travelers to non-MG destinations [7-12]. However, the few studies which have investigated the sources of health advice among travelers to Hajj found that about two-thirds of Hajj pilgrims sought pretravel health advice [13]. In an Australian setting, most Hajj pilgrims (88%) receive the vaccines (eg, meningococcal, influenza, and other travel vaccines) from GPs and a small proportion receive them from other sources including hospitals, workplaces, and travel clinics [13,14]. No study has yet investigated the impact of pretravel health advice on Hajj pilgrims’ awareness of official health recommendations, compliance with preventive measures during Hajj, or incidence of illness symptoms such as cough, sore throat, rhinorrhea, fever, vomiting, and diarrhea. To this end, we conducted a prospective cohort study among Australian Hajj pilgrims, before and during the Hajj in 2015.

## Methods

### Study Design and Targeted Population

Between August and December 2015, a prospective cohort study was conducted among Australian Hajj pilgrims aged ≥18 years planning to attend Hajj 2015, held in the last week of September 2015. Potential participants living in the Greater Sydney region of New South Wales (NSW) were approached. NSW has the largest Muslim population in Australia (50% of the Australian Muslim population), with the majority living in Greater Sydney [15]. Potential participants were approached through their Hajj tour operators during pre-Hajj seminars. As a Hajj requirement, all overseas “would-be” pilgrims must travel on Hajj via an accredited travel agent. The list of accredited Hajj travel agents in Australia, including their addresses, was obtained from the Saudi Arabian Embassy in Canberra, Australia. For accreditation, the travel agents need to demonstrate the ability to organize and manage the Hajj trip, but no formal training is required. Typically, a few months before Hajj, travel agencies run pretravel seminars for the “would be” pilgrims. The frequency, duration, and talk content of these seminars vary by agency: some run several sessions, each lasting one or more days, while others run only one session lasting a few hours. The content of these seminars typically includes spiritual preparation for Hajj, travel itineraries and logistics, and information about the health requirements of the travel, such as vaccinations. The seminars are typically run in a language spoken by the majority of the travelers, or are bilingual.

For this survey, travel agents with the highest quota for Hajj visas were approached first, and the travel agents running their businesses in locations with diverse ethnic groups were prioritized to ensure a diverse sample.

This study was reviewed and approved by the Human Research Ethics Committee (HREC) at The University of Sydney (project number 2014/599).

### Recruitment Methods and Study Variables

#### Overview

This study involved the use of 2 questionnaires: (1) a pre-Hajj questionnaire where the participants were recruited through face-to-face interviews, and (2) self-administrated questionnaires of 6 identical cards of “Hajj” to be completed by the pilgrims daily within a week during the peak Hajj period (from September 21 to 26, 2015). The surveys were primarily in English, and Arabic translations were available for those who preferred it. Data collected before and during Hajj were linked by a unique barcode.

#### Pre-Hajj Survey

The researchers attended 11 seminars held by Hajj travel agents in Sydney from August 1 to September 6, 2015. All attendees at the pre-Hajj seminars were invited to participate, and the surveys were conducted before the seminars to ensure assessment of only pre-existing knowledge. After pilgrims consented to participate in the study, data on their demographic characteristics were obtained using a self-administered questionnaire. Data on the receipt of pretravel health advice were also collected and stratified into 2 major groups: (1) professional medical sources, including advice from GPs and specialist travel clinics; and (2) nonmedical sources, including Hajj travel agencies (tour group leaders), family and friends who had previous experience of Hajj, and the internet. The respondents who obtained advice from professional medical sources were asked about the barriers to receiving pretravel medical advice and their satisfaction regarding the advice they received. The respondents typically completed the questionnaires themselves but the researchers were available on-site, ready to clarify any question that was not clear or to fill out the questionnaire as dictated by the respondent.

#### During Hajj Survey

The researchers travelled to Makkah, Saudi Arabia during the Hajj period and met the study participants (recruited in the pretravel survey) upon their arrival in Mina, Greater Makkah. Each participant was asked to record the following details in the diary (self-reported) for each day: actual use of preventive measures including wearing a face mask, using hand sanitizer (ie, use of soap or alcoholic hand rub), hand washing after touching an ill person, and using disposable handkerchiefs. This diary was completed by the respondents themselves during leisure time; however, a researcher was around to remind them to fill in the questionnaire and provide help if needed. Any respondent who used a preventive measure almost every day (≥5 of 6 days) during the peak Hajj days was considered to be “frequently compliant” with the preventive measure; those who used the preventive measures <5 days were considered to be “infrequently compliant;” and those who did not use the preventive measure at all were considered as “noncompliant.” Self-reported development of symptoms suggestive of a respiratory infection (including cough, sore throat, runny nose, and fever) and other symptoms (including vomiting, diarrhea, and nausea) were also collected. We considered those who reported the presence of a cough, sore throat, and subjective fever to meet the definition of influenza-like illness (ILI) [16].

### Sample Size

A consecutive convenience sampling plan was used to ensure a sample that was representative of Hajj pilgrims residing in NSW. Based on results from our 2014 study [13], and considering an error margin of 5% to be acceptable for this survey, a sample size of 350 pilgrims was deemed sufficient for this survey; this was inflated to 420 to account for loss to follow-up. The targeted sample represented about 12% of Australian pilgrims attending Hajj in 2015.

### Data Analysis

Statistical analysis was performed using SPSS (version 23.0; SPSS Inc). Chi-square tests were used to compare categorical variables. Bivariate analysis with *P* values <.25 were entered into multivariable regression models. Binary logistic regression using the backward Wald method (controlling for factors such as age, gender, chronic medical conditions, educational level, employment status, and undertaken Hajj times) was used to investigate variables related to pretravel health advice–seeking behavior. To study the impact of pretravel health advice sources on pilgrims’ health behaviors (such as face mask use and hand hygiene) and the occurrence of respiratory infections including ILI during Hajj, we compared their behavior according to the most commonly used sources of pretravel health advice among pilgrims: medical (GPs) and nonmedical (Hajj travel agent). We used a logistic regression model (backward Wald method); a two-tailed *P* value of <.05 was considered statistically significant in multivariate models.

## Results

### Overview

A total of 421 pilgrims were recruited before Hajj, and 391 (93%) were followed during Hajj. Of 421 participants aged 18 to 74 (median 41, mean 42.2) years, 54% were male and 28% reported having one or more chronic medical conditions. Over half of participants (225/421, 54%) had up to university level education. In total, 341 pilgrims (81%) were travelling to Hajj for the first time, and pilgrims planned to stay in Saudi Arabia for a median of 25 days (range 10-45 days). Additional participant details are presented in Table 1.

**Table 1:** Demographic characteristics of surveyed participants (N=421).

Demographics				Participants, n (%)
**Gender**				
	Male		229 (54)	
	Female		192 (46)	
**Education level**				
	University level and higher degree		164 (39)	
	Certificate/diploma		61 (15)	
	High school certificate (Year 12 equivalent)		98 (23)	
	School certificate (Year 10 equivalent)		75 (18)	
	No formal education		23 (5)	
**Country of birth**				
	Australia		128 (30)	
	Middle Eastern countries		113 (27)	
	Indian subcontinent		101 (24)	
	Southeast Asian countries		32 (8)	
	Others		42 (10)	
	Median years of stay in Australia^a^		21.5	
**Chronic diseases**				
	No		303 (72)	
	**Yes^b^**		118 (28)	
		Diabetes	41 (35)	
		Asthma	33 (28)	
		High cholesterol	30 (25)	
		Hypertension	28 (24)	
**Overall pretravel health advice–seeking behavior**				
	Sought advice from professional medical health sources		177 (42)	
	Sought advice from nonmedical sources		244 (58)	
**Professional sources**				
	General practitioners		169 (40)	
	Specialist travel clinic		8 (2)	
**Nonprofessional sources**				
	Hajj travel agency		192 (46)	
	Family and friends (who have previous Hajj experience)		38 (9)	
	Internet		14 (3)	

^a^This value only includes those who were born overseas.

^b^Multiple responses were permitted.

### Awareness of Official Hajj Health Recommendations

Over one-third (147/421, 35%) of respondents were aware of the annual Hajj health recommendations issued by the Saudi Arabian MoH; no demographic characteristics were significantly associated with awareness of MoH recommendations. Using multivariable logistic regression analysis, controlling for all other potential variables (pilgrims’ health behaviors including face mask use and hand hygiene with soap and alcoholic hand rub), we found that awareness of official health recommendations was significantly associated only with frequent compliance with hand washing after touching an ill person (adjusted odds ratio [aOR] 2.1, 95% CI 1.1-3.8; *P*=.02).

### Pretravel Advice-Seeking Behavior

All participants obtained some form of pretravel health information before Hajj; in total, 42% (177/421) received pretravel health advice from medical sources and 58% (244/421) received advice solely from nonmedical sources (Table 2).

**Table 2:** Multivariate analysis of the association between receiving pretravel health advice from a medical (general practitioner) or nonmedical source (travel agency) and pilgrims’ health behavior during Hajj.

Health behavior and source of advice		Yes, n (%)	aOR (95% CI)^a,b^	*P* value^a,b^
**Awareness of official Hajj recommendations**				
	General practitioner	76 (52)	0.4 (0.2-0.9)	.03
	Travel agency	98 (67)	2.1 (1.2-3.8)	.01
**Received ≥1 recommended vaccines**				
	General practitioner	177 (54)	0.5 (0.2**-**1.1)	.1
	Travel agency	203 (62)	2.4 (1.4-3.9)	.01
**Face mask use**				
	General practitioner	52 (54)	1.0 (0.4**-**2.1)	.9
	Travel agency	52 (54)	0.6 (0.3**-**1.3)	.2
**Hand washing with soap**				
	General practitioner	170 (50)	1.3 (0.4**-**3.8)	.6
	Travel agency	200 (59)	2.5 (1.1-6.1)	.03
**Hand washing with alcoholic hand rubs**				
	General practitioner	37 (54)	1.8 (0.6**-**5.1)	.2
	Travel agency	39 (57)	1.5 (0.7**-**3.2)	.2
**Hand washing after touching an ill person**				
	General practitioner	33 (56)	1.1 (0.4**-**2.9)	.7
	Travel agency	44 (75)	2.9 (1.1-7.1)	.01
**Use of disposable handkerchiefs**				
	General practitioner	94 (54)	1.1 (0.5**-**2.3)	.6
	Travel agency	97 (56)	0.7 (0.4**-**1.4)	.4

^a^aOR: adjusted odds ratio (binary logistic regression model).

^b^The reference group is the No group (ie, the group who said “no”).

### Professional Medical Sources

In total, 40% (169/421) of participants sought advice from GPs, while 2% (8/421) received advice from a specialized travel clinic. The majority of participants (153/177, 86%) who received professional medical pretravel advice reported that they were satisfied with the advice. In multivariable analysis, those who have diabetes were more likely to receive professional advice (aOR 2.4, 95% CI 1.05-5.9; *P*=.03) than those who did not have diabetes. However, those who were employed were less likely to seek medical pretravel advice than those who were not (aOR 0.5, 95% CI 0.3-0.8; *P*=.01).

Conversely, 58% (244/421) did not seek any professional medical pretravel advice before travelling to Hajj. Reasons for not seeking this advice included the following: preference for other sources (eg, travel agents, friends, and family members; 103/244, 42%), not seeing the need to seek pretravel health advice (96/244; 39%), being too busy (29/244; 12%), and reliance on prior experience or knowledge (16/244; 7%).

### Nonmedical Sources

The most common nonmedical sources pilgrims sought health advice from were Hajj travel agents (192/421; 46%), family and friends who have previous Hajj experience (38/421; 9%), and the internet (14/421, 3%; Table 1).

Multivariable analysis revealed that those who were aged >64 years (aOR 11.1, 95% CI 1.5-81.8; *P*=.01) or employed (aOR 1.5, 95% CI 1.03-2.4; *P*=.03) were more likely to seek advice from nonmedical sources compared to their counterparts.

### The Impact of Pretravel Health Advice on Pilgrims’ Vaccine Uptake, Health Behavior, and the Occurrence of Symptoms During Hajj

Participants who received the recommended vaccines reported various sources of vaccination advice including Hajj travel agents (57%; 189/329), GPs (27%; 90/329), friends and family members with previous Hajj experience (13%; 44/329), and the internet (2%; 6/329). Among all the sources of pretravel health advice, participants who obtained advice from a travel agent were twice as likely to receive the recommended vaccines (aOR 2.4, 95% CI 1.4-3.9; *P*=.01). Additionally, they were more likely to be aware of the official health recommendations (aOR 2.1; 95% CI 1.2-3.8; *P*=.01), wash hands with soap (aOR 2.5, 95% CI 1.1-6.1; *P*=.03), and wash their hands after touching an ill person during Hajj (aOR 2.9, 95% CI 1.1-7.1; *P*=.01) compared to those who sought advice from GPs (Table 2).

In total, 288 (74%) participants reported one or more illness symptoms during Hajj; these were mostly respiratory symptoms, including cough (45%; 176/391), sore throat (44%; 171/391), runny nose (26%; 103/391), and fever (15%; 59/391). ILI was only reported among 10% (40/391) of participants. Nonetheless, the source of the advice was not associated with any reported symptom (Table 3).

**Table 3:** Multivariate analysis of the association between receiving pretravel health advice from a medical (general practitioner) or nonmedical source (travel agency) and the incidence of Hajj-related illness.

Symptom and source of advice		Participants, n (%)	aOR (95% CI)^a,b^	*P* value^a,b^
**Fever**				
	General practitioner	18 (31)	0.8 (0.4-1.6)	.6
	Travel agency	22 (37)	0.7 (0.7-1.4)	.3
**Cough**				
	General practitioner	71 (40)	0.5 (0.2-1.1)	.05
	Travel agency	84 (48)	1.04 (0.6-1.5)	.8
**Sore throat**				
	General practitioner	47 (27)	0.7 (0.3-1.5)	.4
	Travel agency	95 (56)	0.9 (0.6-1.4)	.8
**Runny nose**				
	General practitioner	45 (44)	0.5 (0.2-1.1)	.08
	Travel agency	58 (56)	2.5 (1.1-6.1)	.9
**ILI^c^**				
	General practitioner	17 (41)	0.9 (0.5-1.5)	.7
	Travel agency	21 (52)	0.7 (0.3-1.3)	.3
**Diarrhea**				
	General practitioner	30 (47)	0.7 (0.3-1.8)	.5
	Travel agency	38 (59)	1.5 (0.8-2.8)	.1
**Vomiting**				
	General practitioner	8 (38)	0.8 (0.1-4.1)	.8
	Travel agency	6 (29)	0.5 (0.1-1.6)	.3

^a^aOR: adjusted odds ratio (binary logistic regression model).

^b^The reference group is those who did not seek advice from general practitioners or a travel agency (“No” group).

^c^ILI: influenza-like illness; ILI was defined as cough, sore throat, and subjective fever.

## Discussion

This study shows that travel agencies and GPs were the most commonly sought sources for pretravel advice. Hajj pilgrims who obtained advice from travel agents were more likely to be aware of the official health recommendations, receive recommended vaccines, use hand soaps, and wash their hands after touching an ill person during Hajj compared to those who sought advice from GPs.

This study showed that 42% (177/421) of pilgrims obtained pretravel health advice from professional medical sources; this was somewhat lower than a previous survey among Australian Hajj pilgrims in 2014, which showed that 66% of respondents sought pretravel advice from medical sources [13]. In this study, although GPs were the most commonly sought source of professional advice (169/421, 40%), a small proportion (8/421, 2%) of the respondents sought advice from specialist travel clinics. This contrasts with another Australian survey, which found that 24% of pilgrims sought pretravel advice from specialist travel clinics before Hajj 2014, indicating annual variation or a real decline in the use of specialist travel clinic services [13].

This study found that seeking advice from GPs appeared to have no significant positive impact on vaccination uptake or the use of preventive measures during Hajj. The role of the GP in travel advice is challenging; providing accurate and tailored travel advice during consultations can be affected by limited time and resources. Studies focusing on non–Hajj-related travel found that travel health practitioners and GPs with travel medicine training had higher knowledge of travel advice [17,18], and were more likely to provide written educational materials than primary care physicians without travel medicine training [19,20]. It is noteworthy that in a standard pretravel consultation setting, the common topics of pretravel health advice are travel vaccines (eg, against hepatitis A, typhoid, and yellow fever); malaria prophylaxis; and personal protective measures against insect bites, geographically endemic diseases, food- and water-borne illnesses, and sexually transmitted infections [20-22]. Airborne infections such as influenza, meningococcal disease, and measles, which are the most commonly identified infectious diseases during MGs including Hajj, require a special set of vaccines and preventive measures not typically considered for ordinary travelers [23,24]. Therefore, attempts should be made to encourage GPs and travel practitioners to remain up-to-date with the latest recommendations for specific MGs. This could be achieved by providing accessible educational programs for healthcare providers that are specific to MG travel medicine and coordinated with the timing of events [25]. Uniquely, this study found that not recognizing the need to seek pretravel health advice from medical sources was the main barrier to seeking professional pretravel advice. Therefore, Hajj travelers need to be informed that they need travel health advice and this advice should be sought at least 6 to 8 weeks prior to Hajj [26]. This could be achieved by launching awareness campaigns prior to Hajj about the importance of seeking health advice [27].

This study shows that over half of Australian pilgrims (244/421, 58%) sought health advice from nonprofessional sources, mostly travel agents (192/421, 46%). These results may be due to a high level of confidence in advice from travel agents, and family and friends who had previous Hajj experience, as was also demonstrated in a qualitative study among Australian Hajj pilgrims between 2009 and 2012 [28]. In addition, this study found that receipt of pretravel health advice from specialist travel agents (tour group leaders) was significantly associated with travelers’ health knowledge and behaviors, including being more aware of the health recommendations of the destination country and better compliance with preventive measures. A previous study among Australian Hajj pilgrims in 2014 found that those who obtained health advice from a Hajj travel agency were more likely to be aware of the emerging infectious diseases in Saudi Arabia and receive vaccines than those who did not [13,29]. Similarly, Barasheed et al [14] found that receiving advice from Hajj tour group leaders was the main motivator for the uptake of influenza vaccination among Australian Hajj pilgrims in 2012. Hajj is not the only travel situation where travelers seek advice from tour operators; there are reports of other travelers (eg, tourists, and travelers visiting friends and relatives) seeking advice from travel agents [7-9,11]. Therefore, supplying travel agents with up-to-date, culturally appropriate health information may improve the health awareness and uptake of preventive measures among travelers, including Hajj pilgrims.

In this study, only 35% (147/421) of pilgrims were aware of the annual Hajj health recommendations issued by the Saudi Arabian MoH. Awareness was lower than that found by a previous study that surveyed Australian Hajj pilgrims in 2014, in which 46% of respondents were aware of the health recommendations [13]. Similarly, another study found that only 23% of European attendees at the Union of European Football Associations (UEFA)’s EURO 2012 were aware of the recommendations regarding measles vaccination before the event [30]. This indicates that published official guidelines may not uniformly reach all pilgrims across the world. Importantly, there are no studies that have assessed the usefulness of the official information from health authorities regarding Hajj or any other MGs; however, this study found that awareness of the official health recommendations was, curiously, not associated with pilgrims’ compliance with preventive health measures.

The Saudi Arabian health authority requires that health authorities in pilgrims’ countries of origin provide health education to their pilgrims before the pilgrims travel to Hajj [5]. Several studies found that health education delivered to pilgrims is an effective way of improving their knowledge of infectious diseases as well as the uptake of preventive measures [13,27,31,32]. However, uptake of preventive measures, including vaccination, varies among pilgrims by country of residence [33,34]. For instance, influenza vaccine uptake rates among Australian, French, and Egyptian pilgrims in 2012 were 89%, 46%, and 19.7%, respectively [14,35,36]. Lack of knowledge (particularly of the availability of a vaccine) was the main barrier to vaccine uptake and the use of measures to prevent diseases among pilgrims [13,14,28,37]. Theoretically, health agencies assume that health recommendations will reach Hajj pilgrims through their home country health authority, via health care providers. However, there is no evidence to identify the pathway and link between the issue of the annual Hajj health recommendations from Saudi Arabian health authorities, and how these recommendations are delivered to Hajj pilgrims in their countries. In this study, we identified that Hajj travel agents play an important role in this pathway. More detailed knowledge of this pathway and the dissemination of health advice may improve the promotion of Hajj health recommendations and the uptake of preventive measures among Australian pilgrims.

To our knowledge, this is the first in-depth cohort study investigating the impact of receiving pretravel health advice on travelers’ health behavior during an MG. However, there are some limitations. First, the findings from this survey cannot be widely generalized; the collected data relied on self-reporting and the quality of the health advice could not be evaluated directly. A study of travel agents is needed to complement this study. To this end, we have undertaken a qualitative study among Australian tour operators that assesses their understanding, practice, and advice on infectious disease prevention at Hajj; this study will be reported separately. Second, the “during Hajj” survey, which was conducted consecutively over 6 days by using the same questionnaire diary, may have actually served as a daily reminder for the preventive measures. Third, this being a self-reported survey, it was not possible for us to validate the information the respondents provided; for instance, we could not check if the GPs recorded their pretravel consultations, as is typically done by a trained travel physician. Fourth, it is noteworthy that in this study we could not evaluate the difference in impact between those who sought pre-Hajj advice and those who did not seek advice because all participants obtained some sort of pretravel advice. Finally, travel agents with larger quotas and those with pilgrims from multiple ethnic backgrounds were targeted first, which may have led to some selection bias.

In conclusion, this study has uniquely identified that advice from travel agencies (tour group leaders) reached more travelers than that of GPs or travel health practitioners, and was more strongly associated with travelers’ positive health behaviors. Travel agents are more easily accessible, experienced, inexpensive, and sensitive to culture. However, they do not have specialist medical knowledge and their advice did not appear to result in decreasing the incidence of symptoms of Hajj-related illnesses. This could be potentially addressed by educating agents and tour operators on basic travel health needs.

## Abbreviations

aOR: adjusted odds ratio
GP: general practitioner
ILI: influenza-like illness
MG: mass gathering
MoH: Ministry of Health
NSW: New South Wales
